# Study of tyramine-binding mechanism and insecticidal activity of oil extracted from Eucalyptus against *Sitophilus oryzae*

**DOI:** 10.3389/fchem.2022.964700

**Published:** 2022-09-23

**Authors:** Farshid Zargari, Zahra Nikfarjam, Ebrahim Nakhaei, Masoumeh Ghorbanipour, Alireza Nowroozi, Azam Amiri

**Affiliations:** ^1^ Pharmacology Research Center, Zahedan University of Medical Sciences, Zahedan, Iran; ^2^ Department of Chemistry, Faculty of Science, University of Sistan and Baluchestan (USB), Zahedan, Iran; ^3^ Department of Physical & Computational Chemistry, Chemistry and Chemical Engineering Research Center of Iran, Tehran, Iran; ^4^ Department of Physical Chemistry, Faculty of Chemistry, University of Tabriz, Tabriz, Iran; ^5^ College of Geography and Environmental Planning, University of Sistan and Baluchestan, Zahedan, Iran

**Keywords:** eucalyptus camaldulensis, sitophilus oryzae, GC/MS, tyramine binding mechanism, funnel metadynamics

## Abstract

The rice weevil, Sitophilus oryzae (L.), is a major pest of stored grains throughout the world, which causes quantitative and qualitative losses of food commodities. *Eucalyptus* essential oils (EOs) possess insecticidal and repellent properties, which make them a potential option for insect control in stored grains with environmentally friendly properties. In the current study, the binding mechanism of tyramine (TA) as a control compound has been investigated by funnel metadynamics (FM) simulation toward the homology model of tyramine1 receptor (TyrR) to explore its binding mode and key residues involved in the binding mechanism. EO compounds have been extracted from the leaf and flower part of *Eucalyptus camaldulensis* and characterized by GC/MS, and their effectiveness has been evaluated by molecular docking and conventional molecular dynamic (CMD) simulation toward the TyrR model. The FM results suggested that Asp114 followed by Asp80, Asn91, and Asn427 are crucial residues in the binding and the functioning of TA toward TyrR in Sitophilus Oryzae. The GC/MS analysis confirmed a total of 54 and 31 constituents in leaf and flower, respectively, where most of the components (29) are common in both groups. This analysis also revealed the significant concentration of *Eucalyptus* and α-pinene in leaves and flower EOs. The docking followed by CMD was performed to find the most effective compound in *Eucalyptus* EOs. In this regard, butanoic acid, 3-methyl-, 3-methyl butyl ester (B12) and 2-Octen-1-ol, 3,7-dimethyl- (B23) from leaf and trans- β-Ocimene (G04) from flower showed the maximum dock score and binding free energy, making them the leading candidates to replace tyramine in TyrR. The MM-PB/GBSA and MD analysis proved that the B12 structure is the most effective compound in inhibition of TyrR.

## Graphical Abstract

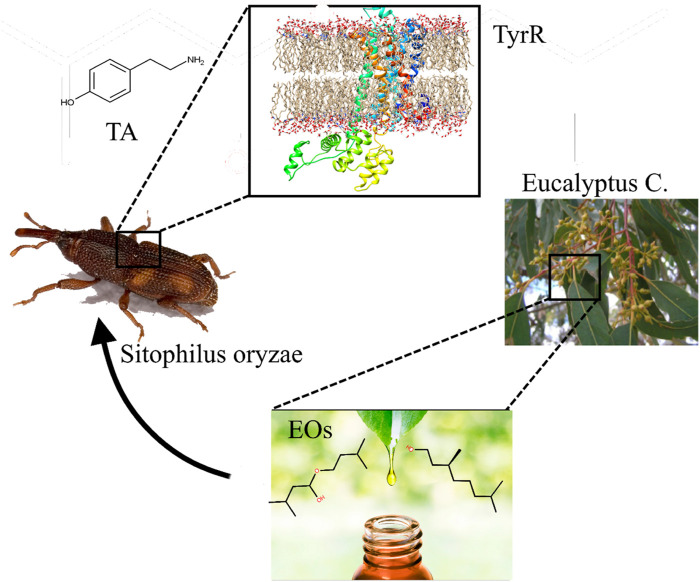

## 1 Introduction

Stored-product insect pests are very popular in cereal industries, as their metabolic wastes and body parts have a detrimental impact on buyer satisfaction (Neethirajan et al., 2007; Chang et al., 2017). It has been reported that 10%–15% of grains are damaged during postharvest in developing countries (Kumar and Kalita, 2017). The rice weevil, Sitophilus oryzae (L.) (Coleoptera: Curculionidae), is a significant insect affecting cereals worldwide (Ahmed, 2001). Its eggs are laid on cereal grain, and the incubated hatchlings dig out a complete grain, where they pupate till they mature into adult weevils (Sharifi and Mills, 1971). Getting into grains of rice, weevil causes quantitative and qualitative alterations and losses of nutritional value and germination, acts as contamination in food commodities with insect bodies and excrement, and most importantly, encourages the growth of storage fungi (Mondal et al., 2016; Seada et al., 2016). While phosphine or even other compounds actually have been employed as fumigants to control rice weevils (Hymavathi et al., 2011), resistance to phosphine administration has been a significant challenge in rice weevil management (Nayak et al., 2007; Hossain et al., 2014). In order to protect stored grain products, additional antiinsect pest techniques are required. Plant-derived essential oils (EOs), derived through nonwoody portions of the plants, mainly foliage, have insecticidal and repellant qualities and can be used to control insects such as Sitophilus oryzae in stored grains (Taylor et al., 2007; Kiran and Prakash, 2015; Hossain et al., 2017). These compositions determine the characteristics of plants and supply plants with a crucial defense plan, especially against herbivorous insect pests and harmful fungus (Dhifi et al., 2016). The genus *Eucalyptus* is one of the most planted species, which include volatile oils in their leaves (Brooker and Kleinig, 1990). For years, essential oils from several *Eucalyptus* species have been employed in the medicinal, beauty, and food fields (Marzoug et al., 2011). Based on previous studies, *Eucalyptus* EO exhibited the highest toxicity to the rice weevil across a variety of EO treatments (Hossain et al., 2019; Ebadollahi and Setzer, 2020). Furthermore, components including 1,8-cineole, citronellal, citronellol, citronellyl acetate, p-cymene, eucamalol, limonene, linalool, -pinene, -terpinene, -terpineol, alloocimene, and aro-madendrene have been linked to the insecticidal activity of Eucalypt (Batish et al., 2008). Among various species of *Eucalyptus*, *Eucalyptus camaldulensis* has the most comprehensive natural distribution, and its essential oils (EOs) have a more complex makeup, with third components accounting for 95% of the total leaf oil found (Dhakad et al., 2018).

According to the literature, the presence of two biogenic amines, octopamine (OA) and tyramine (TA), in high concentrations in the nervous systems of invertebrates shows their pivotal role in neurotransmission, neuromodulation, and neurohormones in insects (Ohta and Ozoe, 2014). As the appearance of octopamine (OctR) and tyramine (TyrR) is limited to invertebrates, two seven-transmembrane protein receptors belonging to a class A G protein-coupled receptor (GPCR) family are the targeting receptors for the introduction of the new bioactive compounds against insects (Degen et al., 2000).

Despite pesticides available for targeting OctR and TyrR in insects (Kostyukovsky et al., 2002), the atomic-level understanding is still in demand to shed light on the OA and TA mechanism of action toward specific target receptors to find and develop new drugs.

Demands for accurate in silico techniques lead researchers to find a visually appealing and cost-effective method to convey valuable, relevant data on protein–ligand binding such as ligand-binding mode, ligand binding free energy, and binding kinetics properties (Broomhead and Soliman, 2017; Raniolo and Limongelli, 2020). Funnel metadynamics (FM) (Limongelli et al., 2013) is a binding free-energy method that attempts to simulate a bias potential flexibly created as a combination of Gaussian functions in the region of chosen degrees of freedom termed collective variables (CVs) to model the binding process of a ligand from its own completely solubilized form to the eventual binding site (Laio and Parrinello, 2002). When the approximate position of the binding site in the protein structure is known but there is little or no information about the ligand-binding mechanism, these strategies come in handy. This method can identify the ligand-binding mode, clarify the dynamics of the ligand-binding mechanism, and calculate the absolute protein–ligand binding free energy (Limongelli et al., 2013; Hsiao and Söderhjelm, 2014; Troussicot et al., 2015; Comitani et al., 2016; Saleh et al., 2017; Saleh et al., 2018; Yuan et al., 2018; Wang et al., 2021). So far, according to the authors’ knowledge, the binding mechanism between TA and Sitophilus oryzae TyrR and the interaction models between this receptor and some monoterpenes have been studied by some researchers (Braza et al., 2019; Ocampo et al., 2020), but there is no systematic approach to this issue. On the other hand, a detailed examination of the interactions between the components of EO *E. camaldulensis* as a bioinsecticide and molecular targets of Sitophilus oryzae has been published; therefore, in the current research, the interaction between *E. camaldulensis* essential oil as a control agent and molecular targets of Sitophilus oryzae as stored product pests has been explored to 1) determine the chemical composition of *E. camaldulensis* EOs, 2) to identify the EO components with the highest affinity to insect molecular targets, and 3) analyze the mechanism of action of more stable EO components on insect molecular targets*.*

## 2 Material and methods

### 2.1 Preparation of e*ucalyptus camaldulensis* material and extraction

First, the aerial parts of *Eucalyptus camaldulensis* (leaf and blossoms) were collected from the botanic farm of the University of Sistan and Baluchestan (USB). Fresh leaves and flowers were disinfected, dried in the sun, and afterward made into a fine powder in a blender. Hydrodistillation with Clevenger (Unividros^®^) equipment and a heated mantle are used to extract the EO. After removing the organic matter, 100 g of plant material was weighed and transferred to a 1 L flask, which was half-filled using distilled water. The extraction took nearly 3 h. Anhydrous sodium sulfate (Na_2_SO_4_, Synth^®^) was used to eliminate trace water from the oil, which was collected in a container. This technique was repeated to extract roughly 3 ml of pure essential oil, and the extracts were subjected to GC/MS studies.

### 2.2 Gas chromatography-mass spectrometry analysis

The analysis of extracted phytochemicals compounds was done with GC-MS (Agilent Technologies 7890B—GC systems 5977A MSD) using the electron impact (EI) mode (ionizing capability 70 eV) and a capillary column (VF-5 ms) (50 m × 0.25 mm, film thickness 0.25 μm) filled with 5% phenyl dimethyl silicone, and the ion supply temperature used was 250°C. In addition, the GC/MS settings are as follows: the preliminary column temperature was set at 35°C and maintained for 5 min; the temperature was increased to 260°C at a rate of 5°C/min, and the split ratio was 1:10. The fraction composition of the samples was computed from the GC peak regions (Supplementary Figures S1, S2). The molecular structure of chemical compounds was approved using the WILEY8, NIST08s, and FAME libraries and is listed in Supplementary Tables S1, S2. The chemical composition of *Eucalyptus camaldulensis* oil revealed the 54/31 constituents for leaves/flowers (Supplementary Figures S3, S4). Among the components of leaves, eucalyptol (22.50%), α-pinene (14.33%), 1H-Cycloprop[e]azulene, decahydro-1,1,7-trimethyl-4-methylene (9.01%), β-pinene (6.32%), and (-)-globulol (5.01%) are the most abundant species, while the major constituents of flowers are eucalyptol (26.5%), α-pinene (16.24%), globulol((-)-globulol) (5.93%), β-pinene (5.80%), and γ-terpinene (5.23%). Evaluating the chemical structure of these species show that most of them (29) are common, while the bicyclo[3.1.0]hex-2-ene,2-methyl-5-(1-methylethyl)- and 1H-Indene,1-ethylideneoctahydro-7a-methyl-,(1E,3aα,7aβ) are only observed in the flower oil. In contrast, all of the other compounds (25) are only related to the oil of leaves.

### 2.3 Homology modeling

It is necessary to find the crystal structure with high-sequence similarity to the TyrR of Sitophilus oryzae in the homology modeling process. The amino acid sequence came from the UniProt database (ID A0A0S1VX60) (Masson et al., 2015). The CLUSTALX program was also used instantly from its website at https://www2.ebi.ac.uk/CLUSTALX to align the sequence of the TyR receptor to that of the D2 dopamine receptor as the template (PDB ID:6CM4) (Thompson et al., 1997). MODELLER (Šali and Blundell, 1993) version 10.1 is used to create homology models of TyrR using the D2 dopamine receptor crystallographic structure and the methods implemented in MODELLER. The 3D models all comprising nonhydrogen atoms were automatically generated from the alignments. The model with the lowermost probability density function (pdf) and the fewest constraint violations was chosen out of 1,000 for further investigation. To improve loops of the chosen model, an ab initio method implemented in the MODELLER was applied. The MODELLER was used to determine the root means square (RMS) deviations of the models concerning the template (6CM4) and determine the R differences using template geometry for bond lengths and angles. MODELLER was also used to determine the R differences using template geometry for bond lengths and angles. The software PROCHECK evaluated the overall stereochemical value of the results and produced a model for each tyramine receptor type (Laskowski et al., 1996). PROCHECK was used to determine the G-factor for the proposed model. In addition, the Verify-3D is also used to validate the environmental profile of the final generated model (Lüthy et al., 1992).

### 2.4 Docking studies

Protein–ligand docking was initiated using LeDock software (http://lephar.com). The initial structure of all compounds, including a total of 54/31 constituents of leaves/flowers that were obtained from gas chromatography-mass spectrometry analysis, was sketched using HyperChem (Teppen, 1992). Then, the geometry optimization and calculation of electronic energy of the benchmark systems were performed using ORCA software (Hočevar and Demšar, 2016) at the DFT, B3LYP/cc-pvdz level of theory. The homology model of Sitophilus oryzae TyrR was selected for docking and subjected to the LePro module (http://lephar.com) for pretreatment of the macromolecule. The docking parameters, including the active site of the protein, were set so that the box with the dimensions of 16 × 16 × 16 Å was placed in the center of D114 and N427, as these are the most critical residues in the active site of TyrR. The number of binding poses and the spacing value are set to 100 and 1.0 Å, respectively. The conformation with the lowest binding energy and the most interacting residues were chosen as the best.

### 2.5 Funnel-metadynamics simulation setup

**FIGURE 1:**
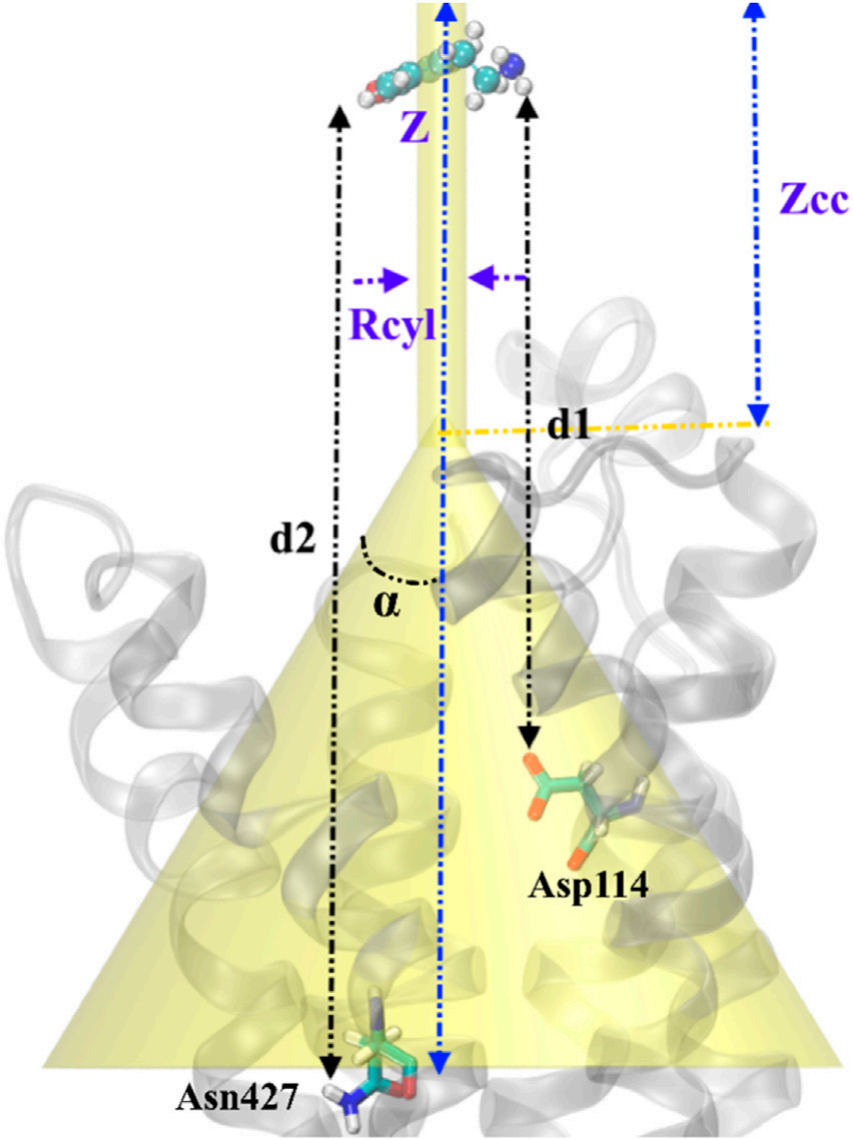
Schematic representation of the funnel restraint potential describes the setup of FM simulation in our work. Binding/unbinding axis, Z defines the binding path of TA from top to inside of transmembrane protein. The distance between cone and cylinder shape is defined as Z_cc_, and R_cyl_ is the radius of the cylindrical section. Two CVs are defined as the distance between the –NH_2_ group in TA and Asp114 (d1) and the distance between the –OH group in TA and Asn427 (d2). TA represents a ball and sticks while the protein shows in the cartoons.

Funnel metadynamics (FM) (Limongelli et al., 2013) simulations were performed using well-tempered metadynamics (Barducci et al., 2008). The funnel parameters are properly defined based on a previous study on GPCRs (Saleh et al., 2017). The PLUMED plugin, the master version (Bonomi et al., 2009), coupled with GROMACS 2020.1 (Pronk et al., 2013), was employed to carry out ∼360 ns of metadynamics simulations in the NPT ensemble. The computational protocol was built by setting the initial Gaussians height at 1.0 kJ/mol and their width at 0.01 Å for the distance between the nitrogen/oxygen atom of tyramine with Asp114 (d1)/TyrR427 (d2) CVs. Gaussians were added every 500 steps (1 ps) so that the deposition rate was equal to 1 kJ/mol·ps. The bias factor was set to 20; consequently, ΔT was 3600 K. The cluster analysis of the conformations found in basin A was performed using the GROMOS algorithm (Daura et al., 1999) of the g-cluster tool implemented in the GROMACS. The absolute TA/TyrR binding free energy was calculated using the following equation (Limongelli et al., 2013):

$$\Delta G_{b}^{0}=-\frac{1}{\beta }\ln\left(k_{b}\right)$$ (1)

where *K*_*b*_ represents the equilibrium binding constant and can be computed as follows:

$$K_{b}=C^{0}\pi R_{cyl}^{2}\int dze^{-\beta \left(w\left(z\right)-w_{ref}\right)}$$ (2)

where C0 is the standard concentration of 1 M and is equal to 1/1.660 Å^−3^ and π $R_{\mathrm{cyl}}^{2}$ is the surface of the cylinder used as a restraint potential in the unbound state. In contrast, the potential W(z) and its value in the unbound state, W_ref_, can be derived from the potential of mean force (PMF) obtained through FM calculations. β is a constant and equal to 1/k_B_T, where k_B_ and T are the Boltzmann constant and the system’s temperature, respectively. Considering cylinder radius R = 1 Å, a schematic of the setup related to the funnel metadynamics is depicted in Figure 1.

### 2.6 Conventional molecular dynamics (CMD) simulations

#### 2.6.1 System setup

Compounds with the best docking pose were chosen to study the interactions of ligands with the active site of the TyrR and to examine the inhibitory efficacy of the ligands. The homology model of TyrR was embedded in the Sphingo and Ceramide Lipid model, which was suggested to be the central part of membrane proteins in insects (Zhou et al., 2013). The membrane was therefore oriented toward the XY plane, bringing the GPCR main axis and the *Z*-axis near to parallel. Also, the VDW and bonded parameters of the TA and the general amber force field (GAFF) were used to detect specified compounds following docking using AmberTools’ antechamber program (Wang et al., 2004), while the protein was modeled by the AMBERff14SB force field (Maier et al., 2015). The partial atomic charges are also calculated by considering the RESP charge model (Vanquelef et al., 2011). The TIP3P water model was used for full solvation, and 0.15 M KCL was employed to neutralize the system. In three dimensions, the periodic boundary condition (PBC) was employed, and all MD simulations were done using a parallel version of SANDER in the AmberTools 19 software package (Case et al., 2018). It is worth noting that, before the MD simulation of protein–ligand complexes, the steepest descent approach was employed to reduce their efficiency and energy, as well as a leap-frog algorithm to integrate their movements (Hockney and Eastwood, 1988). In this procedure, to figure out the effect of long-range electrostatic interactions of molecules, the particle mesh Ewald (PME) method, much like the preceding studies, was implemented (Darden et al., 1993). In addition, the constraints applied on H-bonds using the LINCS algorithm in both equilibration and production run (Hess et al., 1997). The cutoff for nonbonded interactions was set to 12.0 nm. After the optimization of the energy of the system, it was simulated for 200 ps within the canonical ensemble (NVT) and with a 1 ns time-step within the NPT ensemble. Moreover, two models, including the Langevin dynamic model (Goga et al., 2012) and the Parrinello−Rahman one (Parrinello and Rahman, 1981), were served using coupling constants of 0.1 and 0.5 ps to couple the temperature and pressure of the system.

#### 6.2.2 Free energy calculations, energy decomposition, and clustering

Molecular docking is the most popular method in structure-based drug design (Hu and Shelver, 2003), which is applied chiefly to predict the binding pose of candidate drugs in the predefined active site of the protein. However, the accuracy of free energy calculation by docking score might be argued in terms of its reliability in distinguishing between compounds with a comparable binding affinity (Hu and Shelver, 2003).

Among the several methods being used for calculation of binding free energy of ligand–protein complex, the molecular mechanic energies coupled with the Poisson–Boltzmann surface area (MM/PBSA) or generalized Born surface area (MM/GBSA) are proposed in terms of their accuracy and efficacy (Genheden and Ryde, 2012). Here, we used these methods to calculate the relative binding free energies of selected compounds extracted from *Eucalyptus*’s Eos. We used a single MD trajectory of the bound complex in our calculations, and 1,500 snapshots were employed from 15 replicas to estimate the binding free energy of each ligand. To obtain binding free energy of the ligands bound to TyrR, the MMPBSA.Py package has been employed (Miller et al., 2012). We used the modified GB model which is consistent with PB behavior in the electrostatic part of the solvation energy (Feig et al., 2004). The *saltcon* parameter was set to 0.15 M for reconciliation between PB and GB solvation energies, as previously described (Srinivasan et al., 1999).

Decomposition of energy for each residue is defined as the most significant contribution of each residue to the ligand binding, and is classified as the polar, nonpolar, VDW, and electrostatic part of energy for every single residue. We used the water swap residue-wise binding energy decompositions in our work (Kiani et al., 2019).

Clustering of MD frames is, in particular, beneficial for molecular docking simulations. In step with some standards, MD frames that can be positioned within the identical group are just like each other. Consequently, one may want to assume that the alike clusters will behave similarly if a receptor in a cluster interacts agreeably with a selected ligand. The most conventional and regarded degree of similarity is the root mean square deviation (RMSD) values obtained for partitioning MD trajectories, which can be received through pairwise or matrix error distances (De Paris et al., 2015).

### 2.7 Building of the TyrR model and molecular docking

The absence of the crystal structure of TyrR forces us to construct its 3D homology model. Hence, the MODELLER (Šali and Blundell, 1993) was used, employing the D2 Dopamine Receptor as a template to build the structure. It is suggested that structural and sequence similarity within TM regions, in terms of its quality and importance in ligand binding, is preferable to those within intra- or extracellular loops (Mirzadegan et al., 2003; Kinoshita and Okada, 2015).

GMQE (Global Model Quality Estimate) is a quality estimate, which combines properties from the target–template alignment and the template structure. This property for our model is 0.41, which is expected for the model. Despite the low percentage of sequence similarity between target and template (36.7%), it can still be stated that the obtained TyrR model possesses this quality, especially in the transmembrane region where the natural ligand binds (Cavasotto and Phatak, 2009).

**FIGURE 2:**
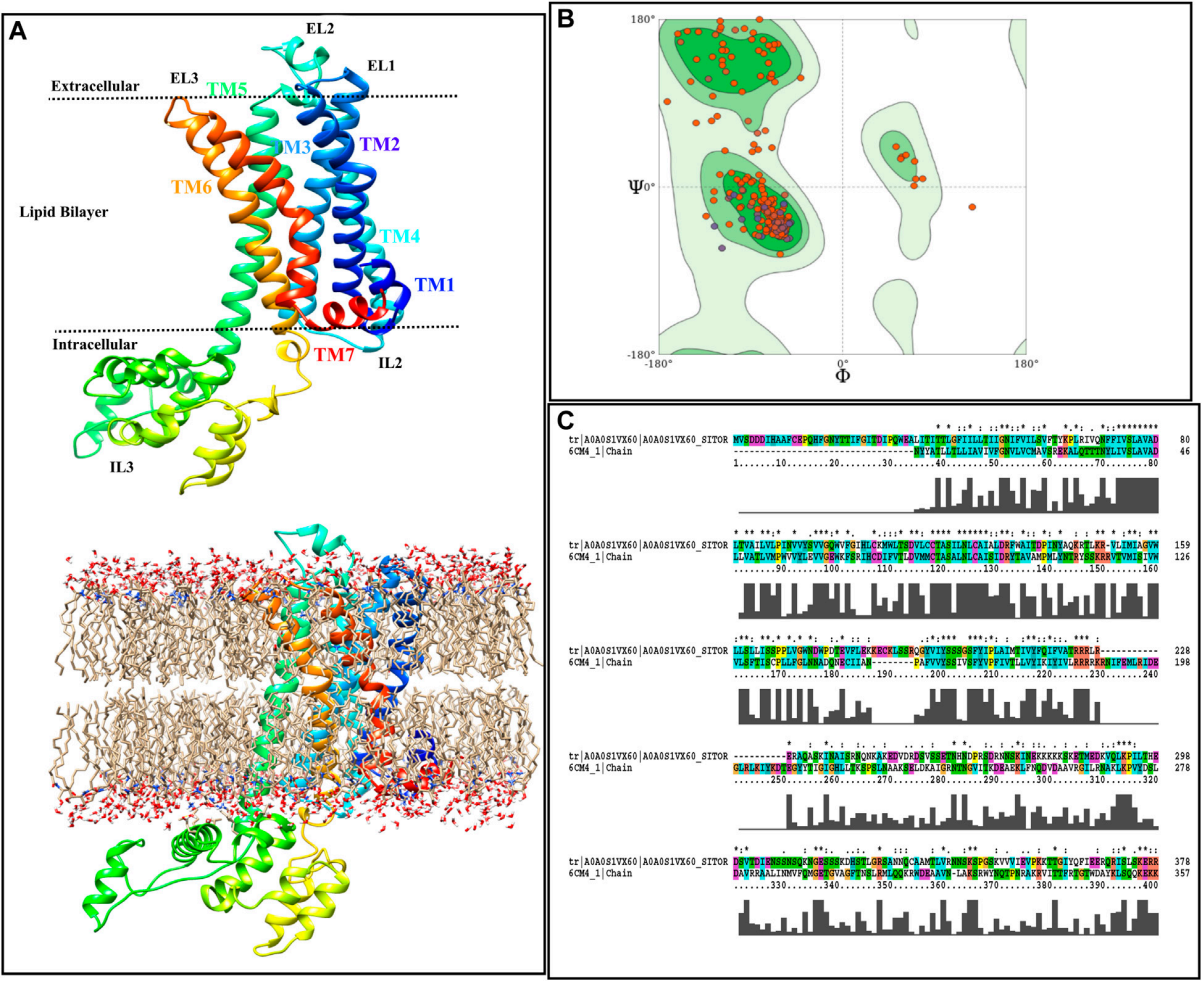
Homology modeling procedure of TyrR is shown. **(A)** The TyrR model describing TM and loop sections and showing the intra/extracellular boundaries of the protein lustration as implicit (top) and explicit (bottom) lipid bilayer, **(B)** Ramachandran diagram is presented to the stereochemical quality of the model made **(C)** alignment of the target sequence to the dopamine D2 receptor with the CLUSTALX program is shown.

Figure 2A illustrates the acquired model annotating TM regions, intra- and extracellular loops, as well as showing the boundaries in which each part is placed. Moreover, the starting model, inserted in the Sphingo and ceramide lipid, constructed by the CHARMM-GUI membrane builder module (Jo et al., 2007), is also represented in Figure 2A. The stereochemical quality of the constructed model is also reported as a Ramachandran plot, and the results are shown in Figure 2B. According to this figure, by the majority of residues in the allowed region, the quality of the model can also be confirmed for further analysis. Figure 2C represents the alignment of the target sequence on the D2 Dopamine Receptor with the CLUSTALX program. The essential residues that play a vital role in the binding of TA in *Bombyx mori*, including Asp114 in TM3, Ser200, and Ser204 in TM5 (Ohta et al., 2004), are conserved in target and template.

Molecular docking is an essential device in structural biology and computer-aided drug design (CADD), in which two molecules fit together in a 3D area [9]. In the present work, the 3D model of TyrR has been constructed as previously described: the active site of the insect’s TyrA receptors including Asp114 residue in TM3 and Ser200 and Ser204 in TM5 (Ohta et al., 2004). The leaf and flower ligands were obtained from PubChem databases and saved in a structure-data file (SDF) format. The ligands were docked onto the TA receptor using LeDock, and the obtained docking energies are depicted in Supplementary Table S1.

According to Supplementary Table S1, the binding affinity of ligands with the receptor active site can be easily discussed by comparing the docking scores. It has been seen that butanoic acid, 3-methyl-, 3-methylbutyl ester (−2.88 kcal/mol), 2-octen-1-ol, 3,7-dimethyl-(-2.86 kcal/mol), citronellol (−2.82 kcal/mol), trans-β-Ocimene (−2.48 kcal/mol), 1,3,6-Octatriene, 3,7-dimethyl-, (Z)- (-2.42 kcal/mol), 1,4-eicosadiene (−2.39 kcal/mol), and 3-eicosyne (−2.14 kcal/mol), respectively, had high binding affinity on the TA receptor than the other compounds in leaf. Also, the docking score of *E. camaldulensis* flower oil structures with TyrR shows that high binding affinity relies upon butanoic acid, 3-methyl-, 3-methyl butyl ester (−2.87 kcal/mol) and β-ocimene (−2.47 kcal/mol) compounds. By taking the docking score of tyramine as a reference binding energy (−2.84 kcal/mol), one can conveniently interpret the binding affinity competition of leaf and flower ligands with TA receptor against tyramine. The results indicate that butanoic acid, 3-methyl-, 3-methylbutyl ester, 2-Octen-1-ol- 3,7-dimethyl-, citronellol, and trans-β-ocimene with the maximum dock score are the main candidates to be replaced instead of tyramine in TA receptor and disrupt its function, the result of may lead to the insect’s death. Finally, for better analyses of these interactions, the 3D structures of TA receptor, tyramine, and the mentioned compounds were selected to proceed toward ligand–protein molecular dynamics studies.

### 2.8 Molecular dynamics simulation

It is important to note that even if based on the analysis of the docking results, it is stated that the ligand is placed in a suitable binding state, it should be kept in mind that in the results obtained from the docking, the effects related to the solvent and temperature are not included. In this regard, the more accurate results related to the binding of the ligand in the activator of the studied protein have been made reliable using MD simulations, and after that, relevant analyses have been performed on the necessary and effective molecular interactions for ligand–protein binding. They also showed the dynamic behavior of the complex at the atomic level in a flexible manner that treated the ligand–receptor complex.

## 3 Result

### 3.1 Identifying the binding pose of TA by FM

**FIGURE 3:**
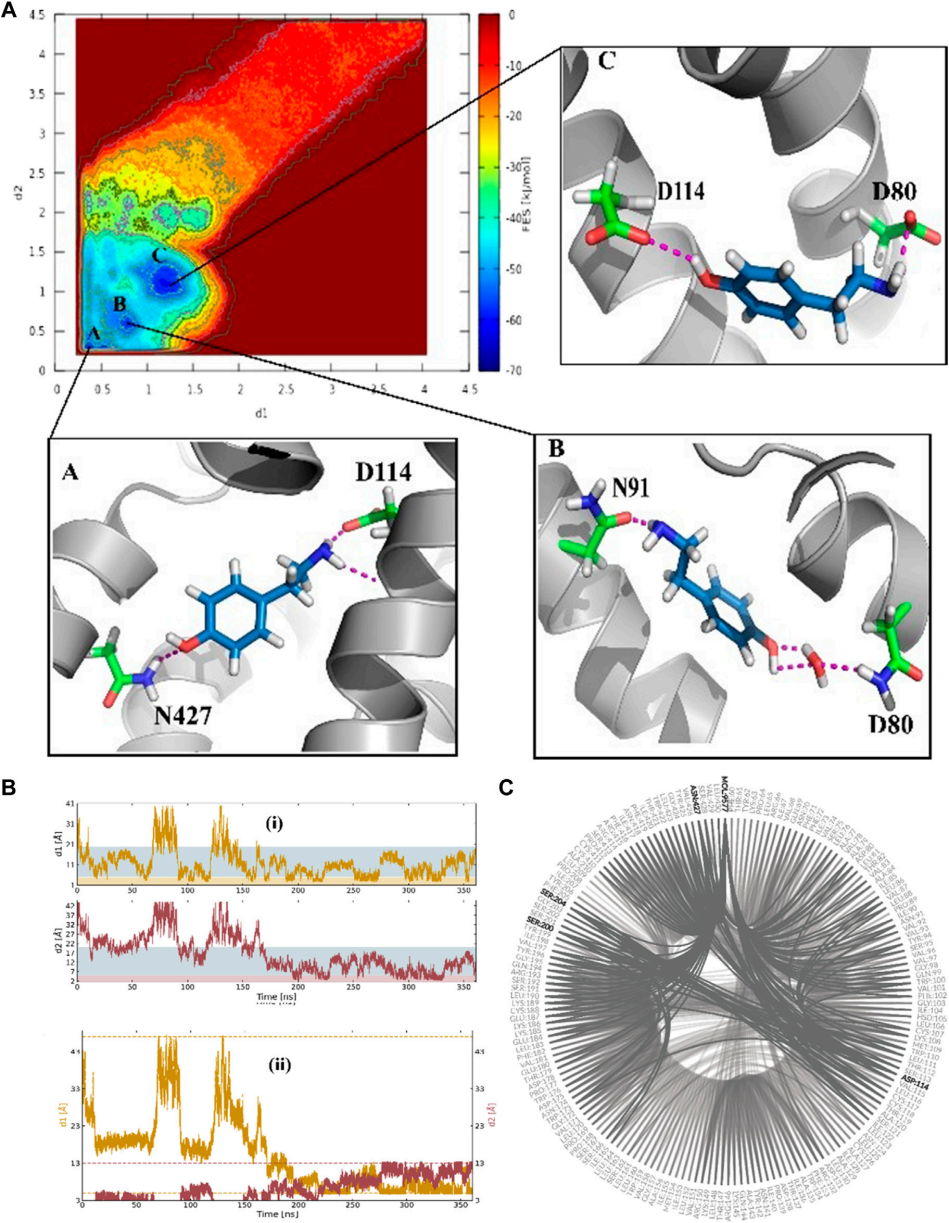
**(A)** The binding energy surface (BFES) of TyrR/TA system showing the location of basins A, B, and C. The binding modes corresponding to each basin are also depicted to show the key residues involved in the binding of TA to its receptor. **(B)** Time evolution of binding/unbinding process during the FM simulation with (i) separate and (ii) amalgamating forms of CVs. The unbound, precomplex, and binding states in (i) are colored as white, blue, and orange, respectively. The recrossing events between bound and unbound states are shown in plot (ii) as a function of simulation time. **(C)** The hydrogen-bonding network (HBN) plot highlighting the key residues during the FM simulation of the TA/TyrR complex.

The funnel-shaped restraining potential was set in a way that its cone was placed on a region surrounding all crucial residues in the proposed binding site to avoid the influence of the restraining potential on the ligand-binding mode. We chose and optimized the dimension for the cone to boost the convergence (Figure 3). As a first choice for the CVs (Figure 3B), we selected the distance between the oxygen atom in TA and the CG atom in Asp114 as d1 and the distance between the nitrogen atom of TA and the ND2 atom in TyrR427 as d2. The convergence was observed after 0.36 μs when the ligand started from the unbound state where it was fully solvated in the water phase, at the extracellular region, and finally found its way to explore the binding site. Several recrossing events were achieved in this trajectory, thereby providing a quantitatively well-characterized FES and an accurate estimation of TyrR-TA binding free energy. The three lowest energy minima (basins) have been detected from the FES corresponding to point A, point B, and point C in Figure 3A. In basin A, TA adopted a configuration in which a hydrogen bond between the –OH group of TA and ND2 atom of Asn427 and two hydrogen bonds between the –NH_2_ group of TA and O and OD atoms of Asp114 occurred. This basin corresponds to the free energy of −62.7 kJ/mol. The configuration of TA in basin C has the same binding energy of −63.0 kJ/mol. The TA is involved in hydrogen bonds between its –OH, –NH_2_ moieties, OD1 atom of Asp114 and OD1 atom of Asp 80, respectively. These findings suggested that Asp 114 is a crucial residue in the binding and the function of TA toward TyrR in Sitophilus Oryzae (Figure 3C).

In basin B, which corresponds to the −58.3 kJ/mol in FES, we observed the water-mediated binding mode, which sheds light on the water’s role in binding structures of TA. In this mode, we can see the hydrogen bond formed between the –NH_2_group of TA and Asn91, a water-mediated hydrogen bond between the –OH moiety in TA and Asp80 (Figure 3C).

### 3.2 TA binding path and evaluating the binding free energy

**FIGURE 4:**
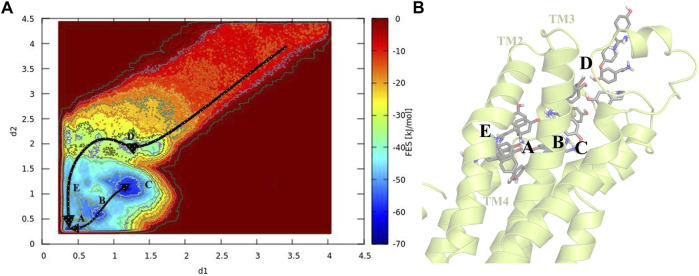
Schematic illustration of the binding pathway trajectory of TA against TyrR is depicted on **(A)** the FES and **(B)** the corresponding 3D structure of the protein. The trajectory showing the binding path is shown as arrows connecting each basin.

To gain a better perception of binding events of TA on the receptor, a rigorous method was required to sample the path of binding/unbinding and produce an exact FES. Hence, we exploited the FM simulation to obtain a quantitatively well-described free energy landscape of ligand binding and calculate the binding free energy of TA against TyrR. In this regard, we track the binding events during the binding process of TA, and the results are depicted in Figure 4. However, as mentioned before in Figure 3A which points to the ligand-binding pathway in reconstruction of a full energy landscape, the arrows are used to illustrate the path constructed by each basin and also the path the ligand adopted during the binding pathway. Figure 4B depicts the frames containing the ligand obtained from the FM trajectory corresponding to each basin in the free energy landscape. The ligand enters when ELs are in the open state (see the next section) and reaches the binding site cleft among the TMs after several binding/unbinding events. In this stage, the ligand dropped in basin D and then tried to find its pathway toward basin E, which corresponds to the cleft between TM2 and TM4. The ligand spent some time in this basin and then found its absolute binding modes corresponding to basins A and C with an intermediate binding mode (basin B), which facilitates the conversion between them (Figure 4).

For the evaluation of the absolute binding free energy of TA, the two minima A and C in FES were considered as bound states, and the ligand at the starting point of the simulation submerging in the balk water was deemed to be the unbound state. The TA binding free energy is calculated initially between these two states. Table 1 represents the binding energy for two basins, A and C, concerning the unbound state, considering the analytical correction.

**TABLE 1:** Affinity and binding free energy of TA against TyrR were obtained from FM simulation and compared with available experimental data. The binding free energies and the binding affinities are calculated for basins A and C.

		Basin A		Basin C	
	IC_50_, _exp_	ΔG_calc_	K_i_^b^	ΔG_calc_	K_i_
TA affinity	5.19^1^	−11.0 ± 0.8	8.64	−11.2 ± 0.8	6.17

Experimental affinity data for *Bombyx mori* was obtained from Ohta et al. (2004) and was converted with the relation Δ*G* = −*RT* ln (*K*_*i*_). ^b^ The IC_50_, K_i_, and *ΔG units are in nM, nM, and kcal.mol* ^*−1*^ *, respectively.*

With the lack of experimental data for the binding affinity of TA toward Sitophilus Oryzae, we used the data for *Bombyx mori* to compare our results to the available experimental data (Ohta et al., 2004). In addition, to provide more structural details on the TA binding, using a reweighting algorithm (Tiwary and Parrinello, 2015), the FES is remapped as a function of the position along the funnel line and distance from the funnel line, producing the FES from the WT-MetaD trajectory above. The WT-MetaD simulations’ binding mechanism is validated mainly through the consistency of the minima found on the two FES (Supplementary Figure S5).

### 3.3 Role of extracellular loops in the binding of TA

**FIGURE 5:**
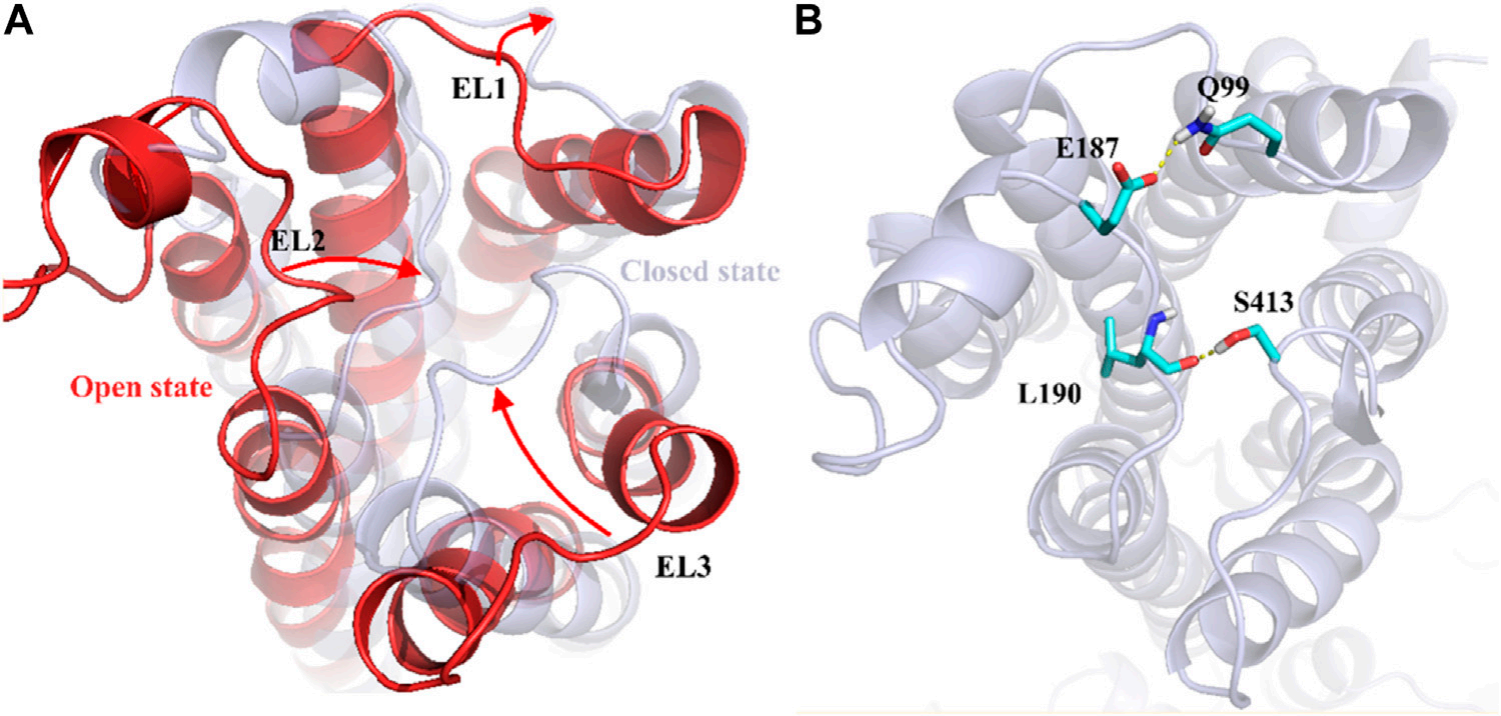
Demonstration of Els’s role in the mechanism of TA binding obtained from FM simulation. **(A)** Transformation of the protein from the open to the closed state, involving EL1, El2, and EL3. The corresponding movement of each EL is depicted as red arrows **(B)** The effective hydrogen bond between residues Glu187 and Gln 99 and also between Leu190 and Ser413, holds EL2 and EL3 together in the closed state.

To understand the physiological action of the TyrR receptor, it is pivotal to characterize the molecular mechanism of TA recognized by the TyrR receptor. The recognition mechanism of peptide and nonpeptide ligands by G protein-coupled receptors (GPCRs) has a different type where peptide ligands prefer to interact primarily with amino acid residues in the extracellular loops (ELs), but nonpeptide ligands such as TA interact predominantly with binding site cleft among the TMs (Baldwin, 1993). Here, we discuss the possible involvement of the Els in the binding mechanism of TA to the TyrR receptor. With a visual inspection, obtained from FM simulation, we observed that the ligand induces conformational changes in ELs at the early stage of approaching the binding site cleft among the TMs. In addition, Figure 5A illustrates the conformational changes in ELs that occur during TA recognition. A moment after entry of ligand to the binding site cleft among TMs, the EL2 and EL3 start to move inside toward the perpendicular axis of the protein. Meanwhile, the EL1 moves outside toward the vertical axis of the protein; these movements change the conformation of the protein from “*open state*” to the “*closed state*,” which curbs the ligand from going back again outside of the protein channel (Figure 5). In the conformation of the protein, changing from an open to a closed state in the TA binding process, we observed that two couples of residues were involved in a strong hydrogen bond to make this conformational change happen. We also showed that the interaction between Glu187 and Gln 99 side chains on the one hand and the hydrogen bond between Leu190 and Ser41, on the other hand, are responsible for keeping EL2 and EL3 close to each other, forming the closed state (Figure 5).

### 3.4 Screening of the EO’s components of *E. camaldulensis*

In the first step of discovering the affinity of essential oil’s components against TyrR and discriminating the effectiveness of compounds in flower and leaf, it is of interest to find the possible binding modes of small molecules in the active site of the protein. The docking was performed as described in the material and method section. To screen all compounds, including 54 in leaf and 31 in flower. The results of the docking are represented in Supplementary Table S1. To make docking results more reliable, it is necessary to evaluate the chosen program in terms of its reproducibility of native ligand (TA) in the TyrR, which is supposed to be the binding mode obtained from FM simulation. Redocking results of TA in the protein have been shown in Supplementary Figure S1A. As shown in the related figure, there is a good match between the LeDock result and the FM binding mode. Therefore, after ensuring the performance of the program, all of the extracted structures, as well as TA, were docked on the active site of the TyrR, the results of which are given in Supplementary Table S1. The two compounds from the leaf and the one from the flower with a high docking score were chosen for further analysis. Figure 5 shows the most effective compounds in the EOs.

### 3.5 Molecular affinity of EO’s components toward TyrR

In this study, three ligand–protein structures have been selected to perform 150 ns of MD simulation, and 1,500 snapshots from 15 replicas have been taken from the MD trajectories to calculate the MM-GB/PBSA binding energies. This may guarantee the accuracy of binding energy obtained from these methods (Sadiq et al., 2010). For this set of ligands, the standard error of the mean provided in this table is expected to be around 1 kcal/mol on average. Table 2 shows the MM-PB/GBSA binding energies for three ligands. The ranks for the abovementioned ligands are demonstrated by the relevant PB/GB binding energies.

**TABLE 2:** Binding free energy and its components obtained by MM-PB/GBSA calculation for all ligands.

		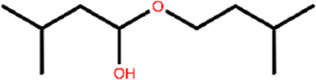	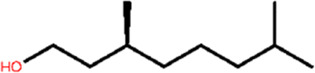	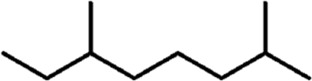
		B12	B23	G04
MM/PBSA	ΔE_VDW_	−24.42	−19.48	−21.66
	ΔE_ele_	−0.64	−1.59	−0.09
	ΔE_PB_	3.96	4.02	2.55
	ΔE_NP_	−21.26	−18.92	−18.44
	ΔG_solv_	20.05	16.89	17.01
	ΔG_gas_	−25.06	−21.07	−21.76
	ΔG_Bind_	−5.01 ± 3.2	−4.17 ± 2.35	−4.74 ± 2.92
MM/GBSA	ΔE_VDW_	−24.42	−19.48	−21.66
	ΔE_elec_	−2.56	−6.37	−0.38
	ΔE_GB_	10.62	14.59	8.88
	ΔE_Surf_	−3.77	−3.20	−3.29
	ΔG_solv_	6.84	11.39	5.58
	ΔG_gas_	−26.99	−25.85	22.04
	ΔG_Bind_	−20.1 ± 3.5	−14.45 ± 3.83	−16.46 ± 2.97

It is of great interest to rank EO’s selected compounds in terms of their binding energy toward the TyrR. According to Table 2, B12 is the most effective compound in the inhibition of the protein. However, it should be noted that the binding energy results are very close to each other, and this indicates that to obtain a more accurate result, further analysis such as decomposition analysis should be performed along with the RMSD, RMSF, and binding modes from cluster analysis and hydrogen bond (H-bond) frequency plots for all three compounds. This result led us to further investigate how B12 could be a viable candidate for inhibiting the protein.

### 3.6 Conformational analysis of the TyrR-EO systems

#### 3.6.1 RMSD analysis

**FIGURE 6:**
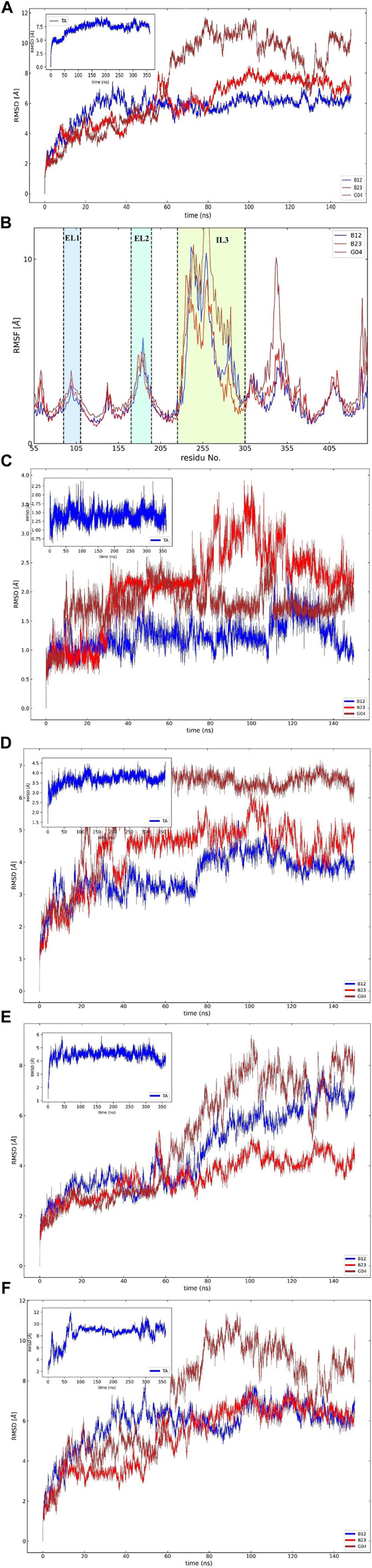
(Continued).

To assess the effective compound in the *Eucalyptus* Camaldulensis EO, we need to inspect the conformational changes of receptors bound to each compound during the MD course and compare them to the conformational pattern we observed from the TA dynamic during FM simulation. The first frame, as a reference conformation, has been used to measure structural changes based on the root mean square deviation (RMSD). We focused on the central regions in the protein whose conformational changes have a significant impact on the function of the protein, that is, transmembrane (TM) helixes and intra-/extracellular (IL/EL) loops. Figure 6A showed the overall RMSD of the protein bound to the EO compounds and TA as a subplot for visual comparison in which, considering the first 150 ns of FM simulation, the B12 and B23 compounds from the flower showed a similar RMSD pattern to TA. We also found that the TMs have negligible contributions to the overall RMSD of the protein due to restricted movement in the membrane bilayer (∼3 Å). Therefore, we concentrated on EL and IL motion to compare the movements of the loops when the compounds B12, B23, and G04 bound to the receptor with those we observed for TA in FM simulation. Figures 6C,D show the RMSD for backbone atoms of EL1 and EL2 loops, respectively. As can be seen, the average RMSD of backbone atoms in EL1 and EL2 in the binding/unbinding process of TA is ∼1.5 Å and 3.5 A, respectively. Among the selected compounds from EO, only B12 shows the same pattern when it binds to the receptor in terms of EL1 and EL2 movements.

The long intracellular loop 3 (IL3) is a 150-amino-acid loop located between the TM5 and TM6 domains. Moreover, research suggested that IL2 and IL3 consist of important interaction areas in GPCRs as well as other cytoplasmic effectors (Gacasan et al., 2017). The RMSD of IL2 and IL3 is given in Figures 6E,F. As can be seen, the flexibilities of IL2 and IL3 have been affected by each EO compound, but it is B12 that asserts the same signal of movements to the IL2 and 3 loops on its binding state.

#### 3.6.2 RMSF values

The influence of screening ligands on the flexibility of the protein structure was studied using root-mean-square fluctuations (RMSFs). Figure 6B shows that three regions, that is, EL1, El2, and IL3, fluctuate the most in the presence of B12, B23, and G04 compounds. B12 and B23 show the nearly same pattern of fluctuation in all regions except IL3.

#### 3.6.3 Clustering analysis

**FIGURE 7:**
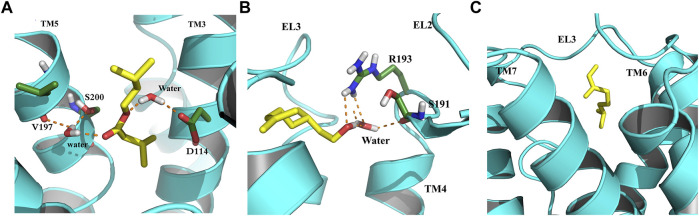
Binding modes of selected EO ligands include **(A)** B12, **(B)** B23, and **(C)** G04 bound to the TyrR after 150 ns of CMD. The protein is represented in cyan, the ligand is yellow, and the hydrogen bonds are represented as orange dashed lines.

In an attempt to elucidate the binding mode of the selected compounds from EOs, the cluster analysis has been done, and the midpoint structure from the most populated cluster has been determined as a representative structure for each ligand–protein complex. Figure 7 shows the representative structures of B12, B23, and G04 bound to TyrR. This can be further evidence for claiming the effectiveness of B12 since, as can be seen in Figure 7A, this ligand is involved in H-bond interactions with Asp114 and Ser200 with water intervention. This is in line with our findings of TA binding mode from FM simulation. The proposed binding modes for B23 and G04 are thoroughly different, indicating different mechanisms of action for these ligands. As it is illustrated in Figure 7B, the B23 involved residues including Arg193 and Ser193 in the EL2 loop. We previously discussed the crucial role of the EL2 loop in the binding of TA, and adaptation of such a binding mode by this ligand could affect the binding mechanism of TA in insects. The same scenario can be imagined for G04 where these ligands also intend to occupy a cleft under the EL3 loop and between TM6 and TM7, as shown in Figure 7C.

#### 3.6.4 Hydrogen bonds analysis

**FIGURE 8:**
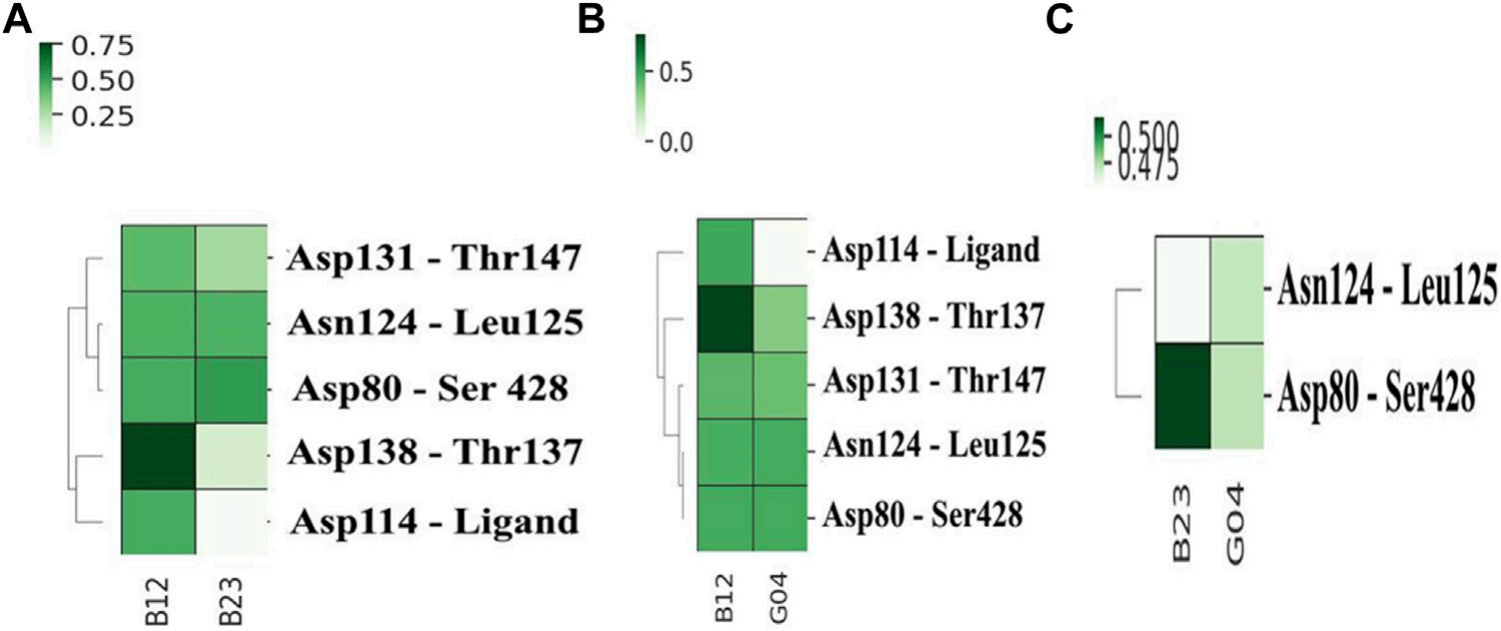
Heat map contacts comparing hydrogen bond formation frequencies in **(A)** B12 and B23, **(B)** B12 and G04, and **(C)** B23 and G04 in the active site of the protein. The related HBN is also shown in each plot.

To compare the stability of each selected ligand, it is a prerequisite to evaluate the contacts it made during the MD simulation. The get contacts (https://getcontacts.github.io/) have been used to make such a comparison between chosen ligands, and the results are shown in Figure 8. In this figure, the most frequent hydrogen bonds are calculated for pairwise combinations of each ligand. These plots not only offer the ligand contacts but also give some information about the hydrogen-bonding network (HBN) in the presence of each ligand. Figure 8A depicts the heat map contacts comparing B12 and B23, and we can see that B12 shows more frequent contacts with Asp114 compared to B23. Likewise, this can be seen in Figures 8B,C, where the heat contact maps compare B12–G04 and B23–G04 in the same fashion, and we see the same trend indicating the effectiveness of B12 involving more hydrogen bonds with critical residues such as Asp114. In addition, we can see some shared contacts in these ligand–protein contact maps, such as Asn124 in contact with Leu125 and Asp80 in contact with Ser428. However, it suggests that the communications between these residues are crucial for HBN and the function of the protein in the presence of these ligands.

#### 3.6.5 Decomposition analysis

**FIGURE 9:**
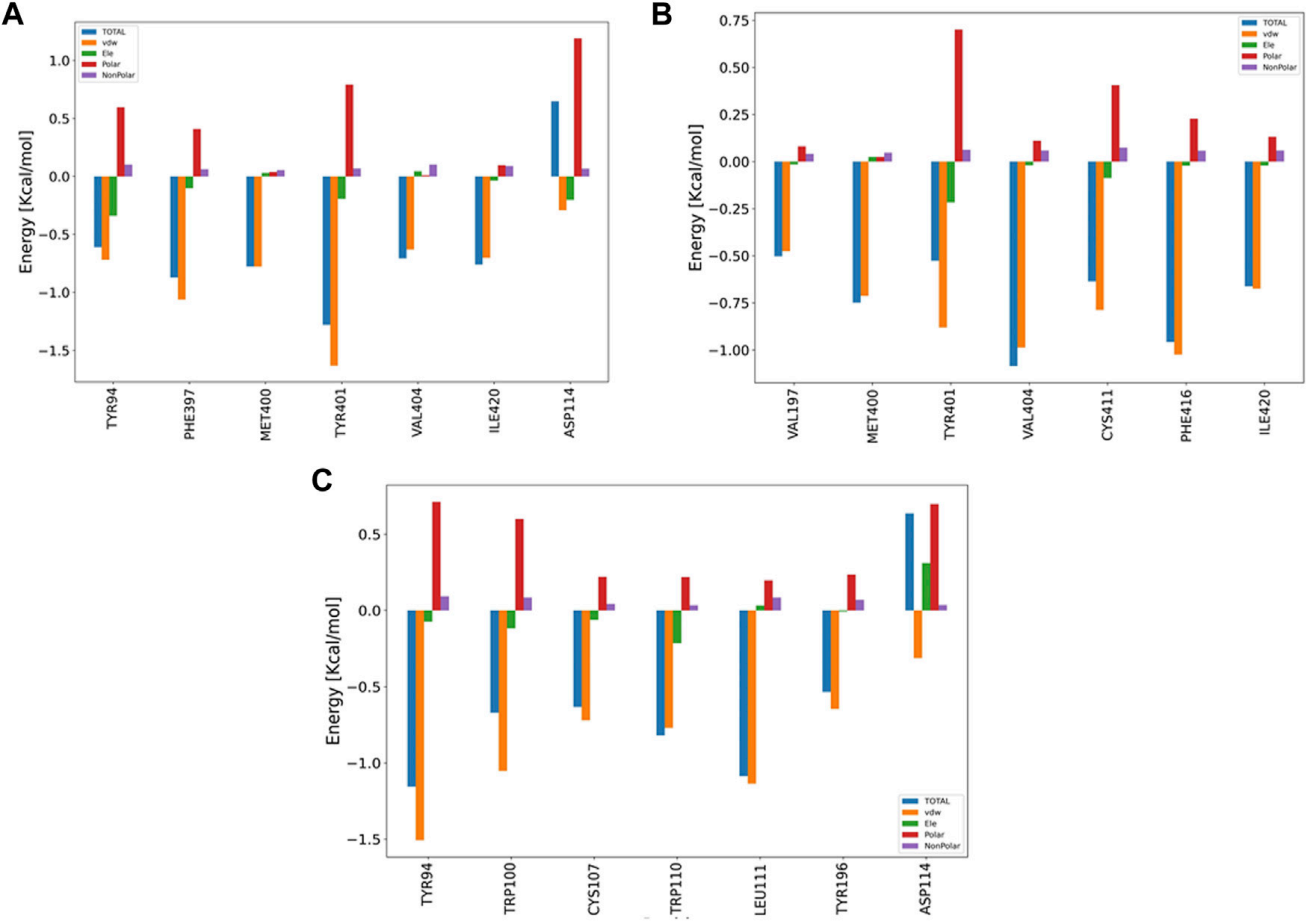
Bar plot depiction of energy decomposition as the VDW, electrostatic, polar solvation, and total energies for B12, B23, and G04.

The pair-wise decomposition analysis can reveal the contribution of energy terms of each residue in the binding energy of the ligand–protein system. Figure 9 illustrates such an analysis for three compounds: B12, B23, and G04. As seen in Figure 9A, we can track down the contribution of Asp114, one of the most essential residues in the binding of TA stressed by experimental and FM simulation, in B12 and G04’s decomposition plots. According to the figure, although the total energy in the decomposition of Asp114 is an adverse effect on the binding energy of B12, the VDW and electrostatic interaction can favor the binding; the necessary information related to the decomposition analysis of B23 and B24 structures are provided in Figures 9B,C, respectively. In the case of G04, as can be seen in Figure 6C, we also observed the contribution of Asp114 in energy binding of this ligand, but lacking a heteroatom in the structure makes it convenient to have merely VDW interaction.

## 4 Discussion

As noted, previous experimental studies showed that *Eucalyptus* essential oil exhibited the highest toxicity to the rice weevil among the variety of EO treatments, and the results show that *E. camaldulensis* essential oils, rich in insecticidal terpenes, can be alternative candidates to synthetic chemicals in the management of S. oryzae. In this regard, the EOs can interfere with neurotransmission by blocking the mechanism of action of OA/TA, which in insects, causes paralysis and may be followed by death (Jankowska et al., 2018). Therefore, in the current study, first, the TA binding mechanism of action toward TyrR has been investigated as a reference to, second, shed light on the interactions between the EO components of *E. camaldulensis* and Sitophilus oryzae tyramine receptor (SoTyrR) with a view toward a detailed analysis of this insecticidal. For this aim, funnel metadynamics and molecular docking, followed by conventional molecular dynamics (CMD) simulation of the ligand–protein complexes, were employed.

In this study, after extracting the EOs from leaves and flowers of *Eucalyptus camaldulensis* using the GC/MS technique, we performed relevant analysis related to the experimental phase. The GC/MS analysis revealed a total of 54/31 constituents for leave/flower chemical composition of *E. camaldulensis* oil, in which most of the components (29) are common. Among the total components, eucalyptol and α-pinene for both chemical groups were the major constituents. Following the experimental phase, computational studies were further investigated. At first, after performing the homology modeling and determining the protein 3D structure with the least error and the most accuracy, the molecular docking method was used to select the appropriate compounds. The docking results show that butanoic acid, 3-methyl-, 3-methylbutyl ester, 2-Octen-1-ol, 3, 7-dimethyl-, citronellol, and trans-β-Ocimene with the maximum dock score are the main candidates to replace instead of tyramine in TA receptor. Free energy methods, which play a pivotal role in drug design research, use two main approaches to calculate free energy. One is to calculate the bound and unbound states separately, in approaches such as the MM/PBSA, and the other is to evaluate the free energy difference between bound and unbound states, which we can term absolute binding free energy. The latter can be executed in two aspects: by decoupling the interactions between the ligand and its receptor, by giving a nonphysical pathway, and by displacing the ligand along a physical pathway of binding. The immediate output of a binding-pathway free energy method is not a free energy difference but a potential of mean force (PMF), which is defined as the negative logarithm of the probability of being at a given value of a specified reaction coordinate (Eqs 1, 2). Funnel metadynamics (FM) is a kind of binding-pathway free energy that calculated the PMF alongside the funnel-shaped pathway.

Therefore, in the current research, using the FM simulation method with high sensitivity and in 360 ns, the mechanism of action and the binding mode of the reference ligand, TA, have been performed. The FM results suggested that Asp114 followed by Asp80, Asn91, and Asn427 are crucial residues in the binding and the function of TA toward TyrR in Sitophilus Oryzae. Finally, in order to explore the effective compounds in EOs, the binding free energies of the selected ligands were investigated from 150 ns of CMD. The two compounds of the leaf (B12 and B23) and the one structure (G04) from a flower with high potential inhibition of the TyrR were chosen for MD analysis. The shreds of evidence from the RMSD, RMSF, hydrogen bonding, clustering, and decomposition analysis indicate that the B12 structure has a higher ability to intervene in the biological function of the TA in the insect.

## Data Availability

The datasets presented in this study can be found in online repositories. The names of the repository/repositories and accession number(s) can be found in the article/Supplementary Material.
