# Supplementary material for: Study of tyramine-binding mechanism and insecticidal activity of oil extracted from Eucalyptus against Sitophilus oryzae
Source: Front Chem. 2022 Sep 23;10:964700. doi: 10.3389/fchem.2022.964700 (PMC9538504; doi:10.3389/fchem.2022.964700)
Supplement: Supplementary file 1 [file DataSheet1.docx]

**Supporting information**

**Study on Tyramine Binding Mechanism and Insecticidal Activity of essential oil from Eucalyptus Camaldulensis on Sitophilus Oryzae**

Farshid Zargari^1,2^, Ebrahim Nakhaei^2^, Zahra Nikfarjam^3^, Masoumeh Ghorbanipour^4^, Alireza Nowroozi^2^

^1^Pharmacology Research Center, Zahedan University of Medical Sciences, Zahedan 9816743463, Iran

^2^Department of Chemistry, Faculty of Science, University of Sistan and Baluchistan (USB), P.O. Box 98135-674, Zahedan, Iran

^3^Department of Molecular and Supramolecular Modelling, Chemistry and Chemical Engineering Research Center of Iran, Tehran, Iran

Department of Physical Chemistry, Faculty of Chemistry, University of Tabriz, Tabriz, Iran^4^

*

| 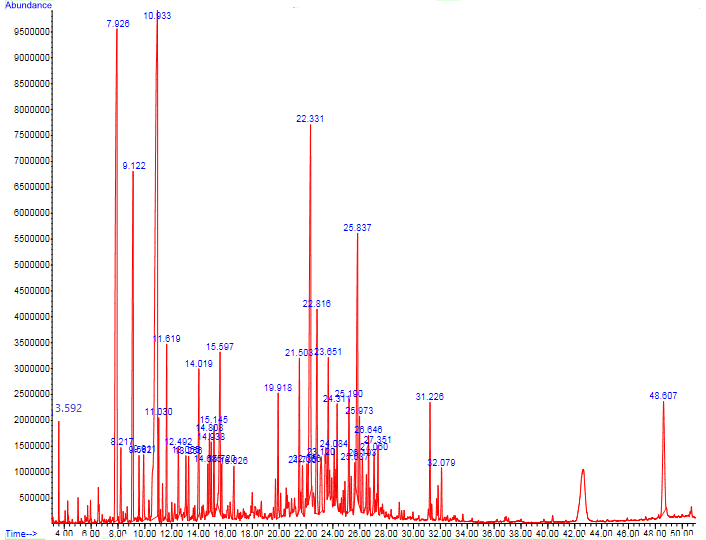 |
| --- |

**Figure S1.** GC-MS chromatogram of E. camaldulensis leaf extract.

| 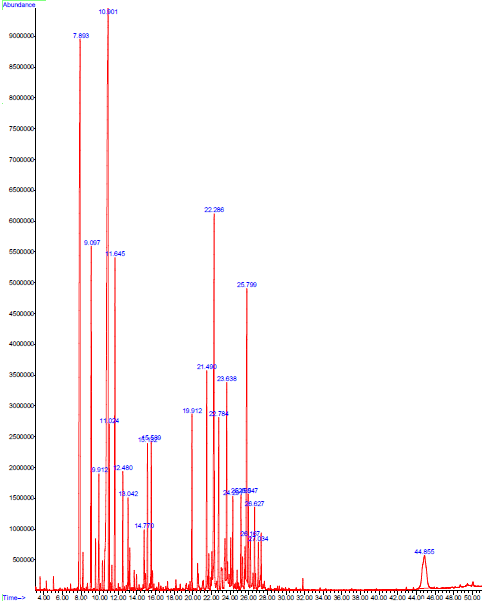 |
| --- |

**Figure S2:** GC-MS chromatogram of E. camaldulensis flowers extract.

| 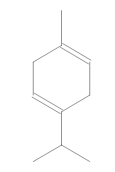 | 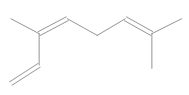 | 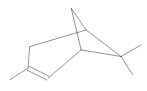 | 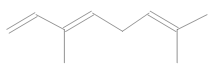 | 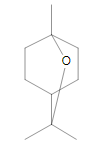 | 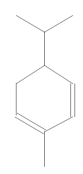 | 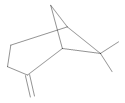 | 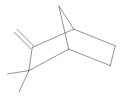 | 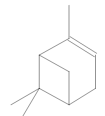 | 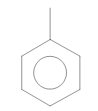 | 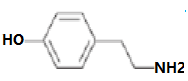 |
| --- | --- | --- | --- | --- | --- | --- | --- | --- | --- | --- |
| γ-Terpinene | 1,3,6-Octatriene, 3,7-dimethyl-, (Z)- | Bicyclo[3.1.1]hept-2-ene, 3,6,6-trimethyl- | β-Ocimene | Eucalyptol | α-Phellandrene | β-Pinene | Camphene | α-Pinene | Toluene | Tyramine(TA) |
| 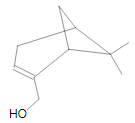 | 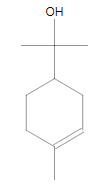 | 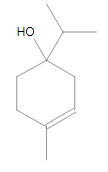 | 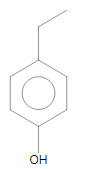 | 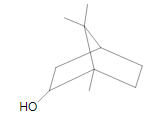 | 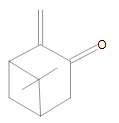 | 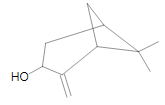 | 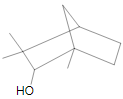 | 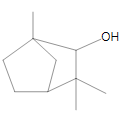 | 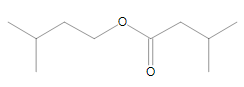 | 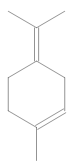 |
| Bicyclo[3.1.1]hept-2-ene-2-methanol, 6,6-dimethyl- | α-Terpineol | Terpinen-4-ol | Phenol, 4-ethyl- | endo-Borneol | Pinocarvone | Bicyclo[3.1.1]heptan-3-ol, 6,6-dimethyl-2-methylene-, [1S-(1α,3α,5α)]- | Bicyclo[2.2.1]  heptan-2-ol, 1,3,3-trimethyl- | Fenchol, exo- | Butanoic acid,  3-methyl-, 3-methylbutyl ester | Cyclohexene, 1-methyl-4-(1-methylethylidene)- |
| 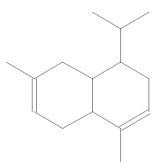 | 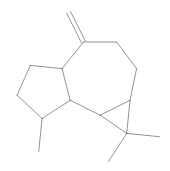 | 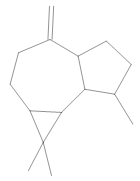 | 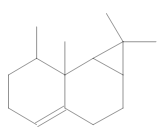 | 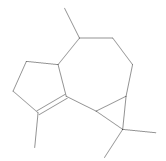 | 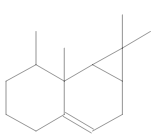 | 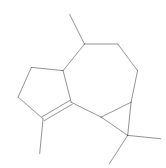 | 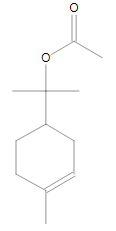 | 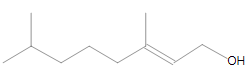 | 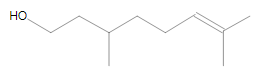 | 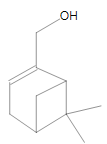 |
| Naphthalene, 1,2,4a,5,8,8a-hexahydro-4,7-dimethyl-1-(1-methylethyl)-, (1α,4aβ,8aα)-(±)- | Aromandendrene | 1H-Cycloprop[e]azulene, decahydro-1,1,7-trimethyl-4-methylene- | 1H-Cyclopropa[a]naphthalene, 1a,2,3,5,6,7,7a,7b-octahydro-1,1,7,7a-tetramethyl-, [1aR-(1aα,7α,7aα,7bα)]- | 1H-Cycloprop[e]azulene, 1a,2,3,4,4a,5,6,7b-octahydro-1,1,4,7-tetramethyl-, [1aR-(1aα,4α,4aβ,7bα)]- | (-)-Aristolene | 1H-Cyclopropa[a]naphthalene, 1a,2,3,3a,4,5,6,7b-octahydro-1,1,3a,7-tetramethyl-, [1aR-(1aα,3aα,7bα)]- | 3-Cyclohexene-1-methanol, α,α,4-trimethyl-, acetate | 2-Octen-1-ol, 3,7-dimethyl- | Citronellol | (-)-Myrtenol |
| 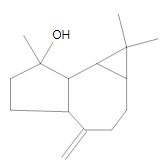 | 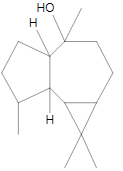 | 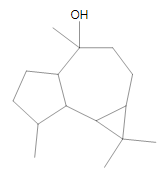 | 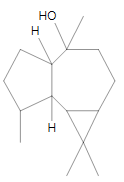 | 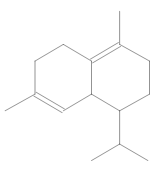 | 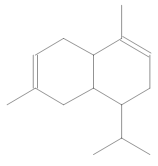 | 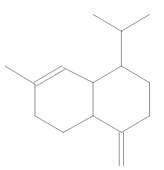 | 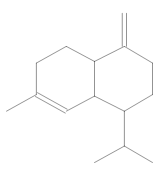 | 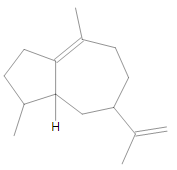 | 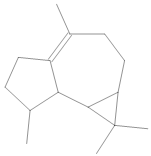 | 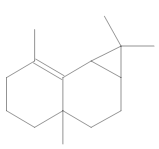 |
| (-)-Spathulenol | Globulol | Ledol | Epiglobulol | Naphthalene, 1,2,3,5,6,8a-hexahydro-4,7-dimethyl-1-(1-methylethyl)-, (1S-cis) | Naphthalene, 1,2,4a,5,8,8a-hexahydro-4,7-dimethyl-1-(1-methylethyl)-, [1S-(1α,4aβ,8aα)] | γ-Muurolene | Naphthalene, 1,2,3,4,4a,5,6,8a-octahydro-7-methyl-4-methylene-1-(1-methylethyl)-, (1α,4aβ,8aα) | Guaia-1(10),11-diene | 1H-Cycloprop[e]azulee, 1a,2,3,5,6,7,7a,7b-octahydro-1,1,4,7-tetramethyl-, [1aR-(1aα,7α,7aβ,7bα)]- | 1H-Cyclopropa[a]naphthalene, 1a,2,3,3a,4,5,6,7b-octahydro-1,1,3a,7-tetramethyl-, [1aR-(1aα,3aα,7bα)]- |
| 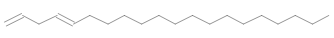 | 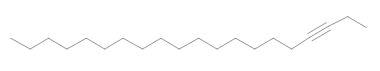 | 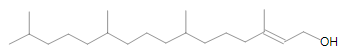 | 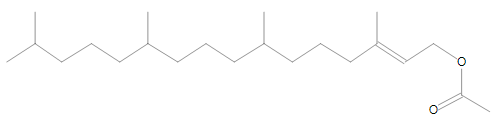 | 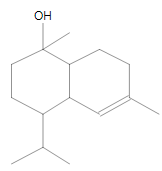 | 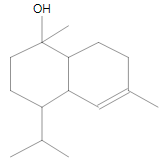 | 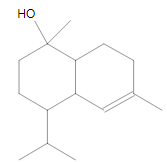 | 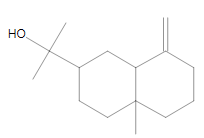 | 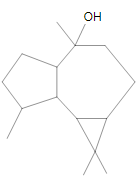 | 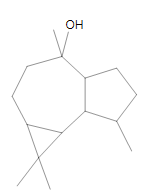 | 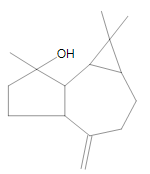 |
| 1,4-Eicosadiene | 3-Eicosyne | 3,7,11,15-Tetramethyl-2-hexadecen-1-ol | Phytol, acetate | tau.-Cadinol | 1-Naphthalenol, 1,2,3,4,4a,7,8,8a-octahydro-1,6-dimethyl-4-(1-methylethyl)-, [1R-(1α,4β,4aβ,8aβ)]-(( α-Cadinol)) | tau.-Muurolol | 2-Naphthalenemethanol, decahydro-α,α,4a-trimethyl-8-methylene-, [2R-(2α,4aα,8aβ)]- | 1H-Cycloprop  [e]azulen-4-ol, decahydro-1,1,4,7-tetramethyl-, [1aR-(1aα,4β,4aβ,7α,7aβ,7bα)]- | (-)-Globulol | 1H-Cycloprop[e]azulen-7-ol, decahydro-1,1,7-trimethyl-4-methylene-, [1ar-(1aα,4aα,7β,7aβ,7bα)]-- |

**Figure S3:** Structures of Tyramine and Chemical composition of E. camaldulensis leaf essential oil

| 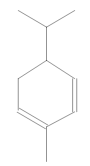 | 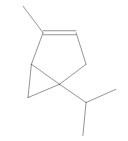 | 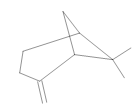 | 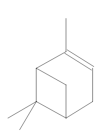 | 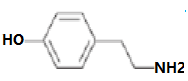 |
| --- | --- | --- | --- | --- |
| α-Phellandrene | Bicyclo[3.1.0]hex-2-ene, 2-methyl-5-(1-methylethyl)- | β-Pinene | α-Pinene | Tyramine (TA) |
| 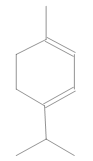 | 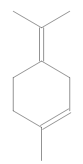 | 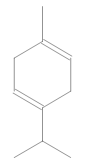 | 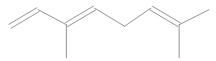 | 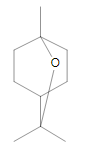 |
| 1,3-Cyclohexadiene, 1-methyl-4-(1-methylethyl)- | Cyclohexene, 1-methyl-4-(1-methylethylidene)- | γ-Terpinene | β-Ocimene | Eucalyptol |
| 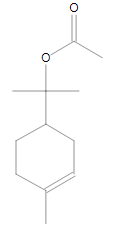 | 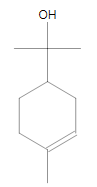 | 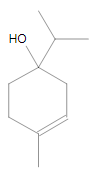 | 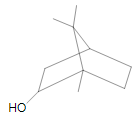 | 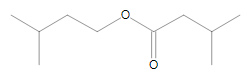 |
| 3-Cyclohexene-1-methanol, α,α,4-trimethyl-, acetate | α-Terpineol | Terpinen-4-ol | endo-Borneol | Butanoic acid, 3-methyl-, 3-methylbutyl ester |
| 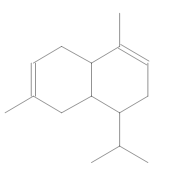 | 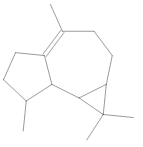 | 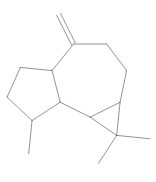 | 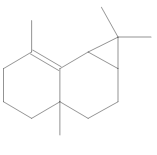 | 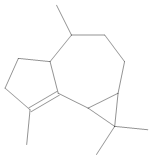 |
| Naphthalene, 1,2,4a,5,8,8a-hexahydro-4,7-dimethyl-1-(1-methylethyl)-, [1S-(1α,4aβ,8aα)]- | 1H-Cycloprop[e]azulene, 1a,2,3,5,6,7,7a,7b-octahydro-1,1,4,7-tetramethyl-, [1aR-(1aα,7α,7aβ,7bα)]- | Alloaromadendrene | 1H-Cyclopropa[a]naphthalene, 1a,2,3,3a,4,5,6,7b-octahydro-1,1,3a,7-tetramethyl-, [1aR-(1aα,3aα,7bα)]- | 1H-Cycloprop[e]azulene, 1a,2,3,4,4a,5,6,7b-octahydro-1,1,4,7-tetramethyl-, [1aR-(1aα,4α,4aβ,7bα)]- |
| 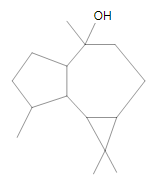 | 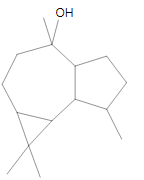 | 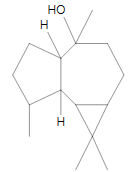 | 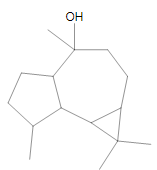 | 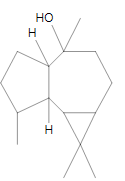 |
| 1H-Cycloprop[e]azulen-4-ol, decahydro-1,1,4,7-tetramethyl-, [1aR-(1aα,4β,4aβ,7α,7aβ,7bα)]- | (-)-Globulol | Globulol | Ledol | Epiglobulol |
| 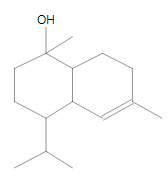 | 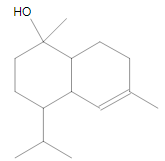 | 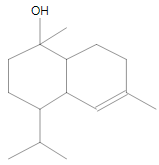 | 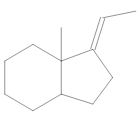 | 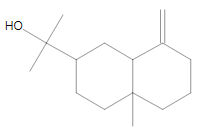 |
| 1-Naphthalenol, 1,2,3,4,4a,7,8,8a-octahydro-1,6-dimethyl-4-(1-methylethyl)-, [1R-(1α,4β,4aβ,8aβ)]- | tau.-Muurolol | tau.-Cadinol | 1H-Indene, 1-ethylideneoctahydro-7a-methyl-, (1E,3aα,7aβ)- | 2-Naphthalenemethanol, decahydro-α,α,4a-trimethyl-8-methylene-, [2R-(2α,4aα,8aβ)]- |
|  |  | 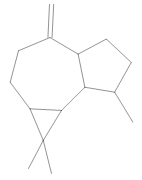 |  |  |
|  |  | 1H-Cycloprop[e]azulene, decahydro-1,1,7-trimethyl-4-methylene- |  |  |

**Figure S4:** Structure of Tyramine,and E. camaldulensis flower oil compounds.

**Table S1.** Chemical composition of E. camaldulensis leaf essential oil, and their score obtained by GC-MS, and docking.

| SCORE  (kcal/mol) | FORMULA | NAME | AREA (%) | R.TIME | PEAK |
| --- | --- | --- | --- | --- | --- |
| -2.84 | C_8_H_11_NO | Tyramine(TA) | - | - | - |
| -1.57 | C_7_H_8_ | Toluene | 0.623 | 3.592 | 1 |
| -1.18 | C_10_H_16_ | α-Pinene | 14.325 | 7.926 | 2 |
| -0.72 | C_10_H_16_ | Camphene | 0.862 | 8.217 | 3 |
| -0.94 | C_10_H_16_ | β-Pinene | 6.315 | 9.122 | 4 |
|  |  |  | 0.600 | 9.562 | 5 |
| -1.50 | C_10_H_16_ | α-Phellandrene | 1.034 | 9.911 | 6 |
| -1.18 | C_10_H_18_O | Eucalyptol | 22.504 | 10.933 | 7 |
| -2.48 | C_10_H_16_ | β-Ocimene |  |  |  |
| -1.09 | C_10_H_16_ | Bicyclo[3.1.1]hept-2-ene, 3,6,6-trimethyl- | 0.824 | 11.030 | 8 |
| -2.42 | C_10_H_16_ | 1,3,6-Octatriene, 3,7-dimethyl-, (Z)- |  |  |  |
| -1.31 | C_10_H_16_ | γ-Terpinene | 1.857 | 11.619 | 9 |
| -1.72 | C_10_H_16_ | Cyclohexene, 1-methyl-4-(1-methylethylidene)- | 0.947 | 12.492 | 10 |
| -2.88 | C_10_H_20_O_2_ | Butanoic acid, 3-methyl-, 3-methylbutyl ester | 0.690 | 13.055 | 11 |
| -1.48 | C_10_H_18_O | Fenchol, exo- | 0.742 | 13.256 | 12 |
| -1.45 | C_10_H_18_O | Bicyclo[2.2.1]heptan-2-ol, 1,3,3-trimethyl- |  |  |  |
| -1.19 | C_10_H_16_O | Bicyclo[3.1.1]heptan-3-ol, 6,6-dimethyl-2-methylene-, [1S-(1α,3α,5α)]- | 2.023 | 14.019 | 13 |
| -1.25 | C_10_H_14_O | Pinocarvone | 0.508 | 14.685 | 14 |
| -1.29 | C_10_H_18_O | endo-Borneol | 1.019 | 14.808 | 15 |
| -2.06 | C_8_H_10_O | Phenol, 4-ethyl- | 0.667 | 14.938 | 16 |
| -1.71 | C_10_H_18_O | Terpinen-4-ol | 1.042 | 15.145 | 17 |
| -1.90 | C_10_H_18_O | α-Terpineol | 2.771 | 15.597 | 18 |
| -1.64 | C_10_H_16_O | Bicyclo[3.1.1]hept-2-ene-2-methanol, 6,6-dimethyl- | 0.639 | 15.720 | 19 |
| -1.70 | C_10_H_16_O | (-)-Myrtenol |  |  |  |
| -2.82 | C_10_H_20_O | Citronellol | 0.832 | 16.626 | 20 |
| -2.86 | C_10_H_20_O | 2-Octen-1-ol, 3,7-dimethyl- |  |  |  |
| -1.70 | C_12_H_20_O_2_ | 3-Cyclohexene-1-methanol, α,α,4-trimethyl-, acetate | 1.545 | 19.918 | 21 |
| -0.54 | C_15_H_24_ | 1H-Cyclopropa[a]naphthalene, 1a,2,3,3a,4,5,6,7b-octahydro-1,1,3a,7-tetramethyl-, [1aR-(1aα,3aα,7bα)]- | 1.997 | 21.503 | 22 |
| -0.39 | C_15_H_24_ | (-)-Aristolene | 0.672 | 21.736 | 23 |
| -0.55 | C_15_H_24_ | 1H-Cycloprop[e]azulene, 1a,2,3,4,4a,5,6,7b-octahydro-1,1,4,7-tetramethyl-, [1aR-(1aα,4α,4aβ,7bα)]- | 0.553 | 22.066 | 24 |
| -0.40 | C_15_H_24_ | 1H-Cyclopropa[a]naphthalene, 1a,2,3,5,6,7,7a,7b-octahydro-1,1,7,7a-tetramethyl-, [1aR-(1aα,7α,7aα,7bα)]- |  |  |  |
| -0.60 | C_15_H_24_ | 1H-Cycloprop[e]azulene, decahydro-1,1,7-trimethyl-4-methylene- | 9.010 | 22.331 | 25 |
| -0.58 | C_15_H_24_ | Aromandendrene | 3.414 | 22.816 | 26 |
| -0.89 | C_15_H_24_ | Naphthalene, 1,2,4a,5,8,8a-hexahydro-4,7-dimethyl-1-(1-methylethyl)-, (1α,4aβ,8aα)-(±)- | 1.506 | 23.120 | 27 |
| -0.51 | C_15_H_24_ | 1H-Cyclopropa[a]naphthalene, 1a,2,3,3a,4,5,6,7b-octahydro-1,1,3a,7-tetramethyl-, [1aR-(1aα,3aα,7bα)]- |  |  |  |
| -0.64 | C_15_H_24_ | 1H-Cycloprop[e]azulee, 1a,2,3,5,6,7,7a,7b-octahydro-1,1,4,7-tetramethyl-, [1aR-(1aα,7α,7aβ,7bα)]- | 1.691 | 23.651 | 28 |
| -0.76 | C_15_H_24_ | Guaia-1(10),11-diene |  |  |  |
| -0.86 | C_15_H_24_ | Naphthalene, 1,2,3,4,4a,5,6,8a-octahydro-7-methyl-4-methylene-1-(1-methylethyl)-, (1α,4aβ,8aα)- | 0.597 | 24.084 | 29 |
| -0.80 | C_15_H_24_ | γ-Muurolene |  |  |  |
| -0.77 | C_15_H_24_ | Naphthalene, 1,2,4a,5,8,8a-hexahydro-4,7-dimethyl-1-(1-methylethyl)-, [1S-(1α,4aβ,8aα)]- | 1.023 | 24.311 | 30 |
| -0.81 | C_15_H_24_ | Naphthalene, 1,2,3,5,6,8a-hexahydro-4,7-dimethyl-1-(1-methylethyl)-, (1S-cis)- |  |  |  |
| -0.67 | C_15_H_26_O | Epiglobulol |  |  |  |
| -0.73 | C_15_H_26_O | Ledol | 1.586 | 25.190 | 31 |
| -0.55 | C_15_H_26_O | Globulol |  |  |  |
| -0.73 | C_15_H_24_O | (-)-Spathulenol | 0.557 | 25.637 | 32 |
| -0.68 | C_15_H_24_O | 1H-Cycloprop[e]azulen-7-ol, decahydro-1,1,7-trimethyl-4-methylene-, [1ar-(1aα,4aα,7β,7aβ,7bα)]- |  |  |  |
| -0.74 | C_15_H_26_O | (-)-Globulol | 5.008 | 25.837 | 33 |
| -0.79 | C_15_H_26_O | 1H-Cycloprop[e]azulen-4-ol, decahydro-1,1,4,7-tetramethyl-, [1aR-(1aα,4β,4aβ,7α,7aβ,7bα)]- | 1.244 | 25.973 | 34 |
| -1.21 | C_15_H_26_O | 2-Naphthalenemethanol, decahydro-α,α,4a-trimethyl-8-methylene-, [2R-(2α,4aα,8aβ)]- | 0.761 | 26.193 | 35 |
|  |  |  | 0.833 | 26.646 | 36 |
| -0.59 | C_15_H_26_O | tau.-Muurolol | 0.913 | 27.060 | 37 |
| -0.67 | C_15_H_26_O | 1-Naphthalenol, 1,2,3,4,4a,7,8,8a-octahydro-1,6-dimethyl-4-(1-methylethyl)-, [1R-(1α,4β,4aβ,8aβ)]-(( α-Cadinol)) |  |  |  |
| -0.79 | C_15_H_26_O | tau.-Cadinol | 0.593 | 27.351 | 38 |
| -0.97 | C_22_H_42_O_2_ | Phytol, acetate |  |  |  |
| -1.90 | C_20_H_40_O | 3,7,11,15-Tetramethyl-2-hexadecen-1-ol | 1.293 | 31.226 | 39 |
| -2.14 | C_20_H_38_ | 3-Eicosyne |  |  |  |
| -2.39 | C_20_H_38_ | 1,4-Eicosadiene | 0.668 | 32.079 | 40 |

**Table S2.** Compounds of E. camaldulensis flower oil obtained by GC MS reports, and their score docking.

| SCORE  (kcal/mol) | FORMULA | NAME | AREA  (%) | R.TIME | PEAK |
| --- | --- | --- | --- | --- | --- |
| -2.84 | C_8_H_11_NO | Tyramine (TA) | - | - | - |
| -1.21 | C_10_H_16_ | α-Pinene | 16.236 | 7.893 | 1 |
| -0.93 | C_10_H_16_ | β-Pinene | 5.802 | 9.097 | 2 |
| -1.35 | C_10_H_16_ | Bicyclo[3.1.0]hex-2-ene, 2-methyl-5-(1-methylethyl)- | 1.523 | 9.912 | 3 |
| -1.51 | C_10_H_16_ | α-Phellandrene |  |  |  |
| -1.19 | C_10_H_18_O | Eucalyptol | 26.512 | 10.901 | 4 |
| -2.47 | C_10_H_16_ | β-Ocimene |  |  |  |
|  |  |  | 1.808 | 11.024 | 5 |
|  |  |  |  |  |  |
| -1.30 | C_10_H_16_ | γ-Terpinene | 5.229 | 11.645 | 6 |
| -1.73 | C_10_H_16_ | Cyclohexene, 1-methyl-4-(1-methylethylidene)- | 1.445 | 12.480 | 7 |
| -1.45 | C_10_H_16_ | 1,3-Cyclohexadiene, 1-methyl-4-(1-methylethyl)- |  |  |  |
| -2.87 | C_10_H_20_O_2_ | Butanoic acid, 3-methyl-, 3-methylbutyl ester | 0.987 | 13.042 | 8 |
| -1.24 | C_10_H_18_O | endo-Borneol | 0.845 | 14.770 | 9 |
| -1.68 | C_10_H_18_O | Terpinen-4-ol | 2.049 | 15.132 | 10 |
| -1.88 | C_10_H_18_O | α-Terpineol | 2.078 | 15.539 | 11 |
| -1.86 | C_12_H_20_O2 | 3-Cyclohexene-1-methanol, α,α,4-trimethyl-, acetate | 2.449 | 19.912 | 12 |
| -0.54 | C_15_H_24_ | 1H-Cycloprop[e]azulene, 1a,2,3,4,4a,5,6,7b-octahydro-1,1,4,7-tetramethyl-, [1aR-(1aα,4α,4aβ,7bα)]- | 3.176 | 21.490 | 13 |
| -0.54 | C_15_H_24_ | 1H-Cyclopropa[a]naphthalene, 1a,2,3,3a,4,5,6,7b-octahydro-1,1,3a,7-tetramethyl-, [1aR-(1aα,3aα,7bα)]- |  |  |  |
| - | - | - | 7.461 | 22.286 | 14 |
| -0.57 | C_15_H_24_ | Alloaromadendrene | 2.858 | 22.784 | 15 |
| -0.66 | C_15_H_24_ | 1H-Cycloprop[e]azulene, 1a,2,3,5,6,7,7a,7b-octahydro-1,1,4,7-tetramethyl-, [1aR-(1aα,7α,7aβ,7bα)]- | 2.818 | 23.638 | 16 |
| -0.83 | C_15_H_24_ | Naphthalene, 1,2,4a,5,8,8a-hexahydro-4,7-dimethyl-1-(1-methylethyl)-, [1S-(1α,4aβ,8aα)]- | 1.069 | 24.291 | 17 |
| -0.67 | C_15_H_26_O | Epiglobulol |  |  |  |
| -0.74 | C_15_H_26_O | Ledol | 1.371 | 25.165 | 18 |
|  |  |  |  |  |  |
| -0.75 | C_15_H_26_O | Globulol | 5.938 | 25.799 | 19 |
| -0.74 | C_15_H_26_O | (-)-Globulol |  |  |  |
| -0.77 | C_15_H_26_O | 1H-Cycloprop[e]azulen-4-ol, decahydro-1,1,4,7-tetramethyl-, [1aR-(1aα,4β,4aβ,7α,7aβ,7bα)]- | 1.520 | 25.947 | 20 |
| -1.23 | C_15_H_26_O | 2-Naphthalenemethanol, decahydro-α,α,4a-trimethyl-8-methylene-, [2R-(2α,4aα,8aβ)]- | 0.863 | 26.167 | 21 |
| -1.04 | C_12_H_20_ | 1H-Indene, 1-ethylideneoctahydro-7a-methyl-, (1E,3aα,7aβ)- | 1.025 | 26.627 | 22 |
| -0.82 | C_15_H_26_O | tau.-Cadinol |  |  |  |
| -0.48 | C_15_H_26_O | tau.-Muurolol | 0.803 | 27.034 | 23 |
| -0.56 | C_15_H_26_O | 1-Naphthalenol, 1,2,3,4,4a,7,8,8a-octahydro-1,6-dimethyl-4-(1-methylethyl)-, [1R-(1α,4β,4aβ,8aβ)]- |  |  |  |
| -0.54 | C_15_H_24_ | 1H-Cycloprop[e]azulene, decahydro-1,1,7-trimethyl-4-methylene- | 4.135 | 44.855 | 24 |


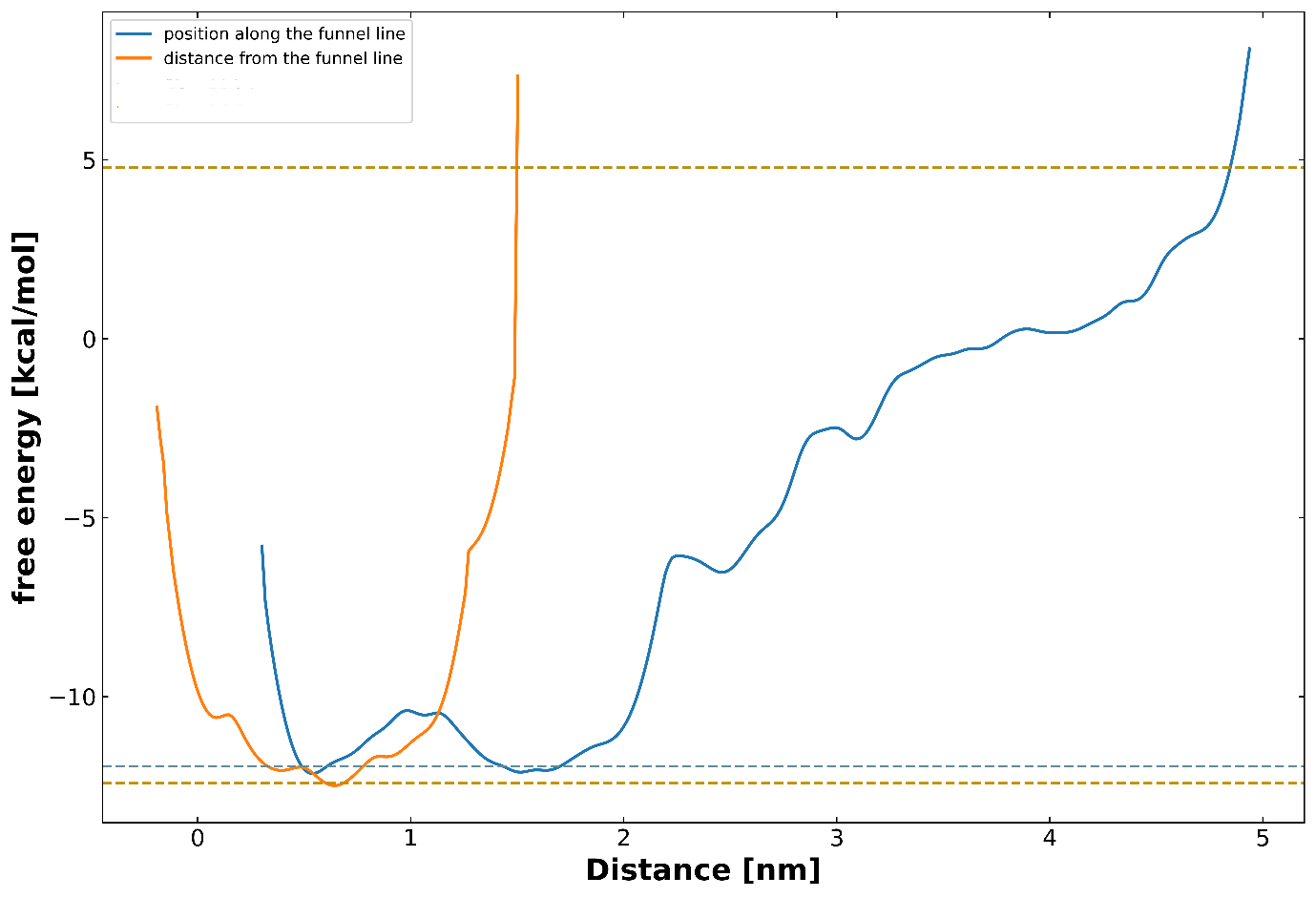


**Figure S5:** Reweighting of the FM simulation as a function of the position along the funnel line and distance from the funnel line.
